# Evidence of population expansion and insecticide resistance mechanism in invasive fall armyworm (*Spodoptera frugiperda*)

**DOI:** 10.1186/s12896-023-00786-6

**Published:** 2023-07-04

**Authors:** Snigdha Samanta, Mritunjoy Barman, Himanshu Thakur, Swati Chakraborty, Gouranga Upadhyaya, Deepayan Roy, Amitava Banerjee, Arunava Samanta, Jayanta Tarafdar

**Affiliations:** 1 Department of Agricultural Entomology, Bidhan Chandra Krishi Viswavidyalaya, Mohanpur, West Bengal India; 2 School of Agriculture Science, GD Goenka University, Gurugram, Haryana 122103 India; 3 Department of Entomology, C.S.K. Himachal Pradesh Krishi Vishvavidyalaya, Palampur, Himachal Pradesh India; 4 Department of Plant Pathology, Bidhan Chandra Krishi Viswavidyalaya, Mohanpur, West Bengal India; 5 Department of Biological Sciences, Indian Institute of Science Education and Research Kolkata, Kolkata, West Bengal 741246 India

**Keywords:** Insecticide resistance, *COI* marker, Genetic diversity, Fall Armyworm

## Abstract

**Background:**

The invasive and calamitous polyphagous pest *Spodoptera frugiperda* or commonly known as fall armyworm (FAW) poses serious menace to the global agricultural production. Owing to the revamped invasion of FAW in 2018 in India, present study was undertaken for precise assessment of its genetic identity and pesticide resistance to aid in pest-management strategies.

**Results:**

To evaluate the diversity in FAW population across Eastern India, mitochondrial *COI* sequences were used which revealed a low nucleotide diversity. Analysis of molecular variance indicated significant genetic variation between four global geographical FAW populations with lowest differentiation between India and Africa suggesting a present-day and shared origin of FAW. The study demonstrated existence of two different strains (‘R’ strain and ‘C’ strain) based on *COI* gene marker. However, discrepancies between *COI* marker and host plant association of FAW was observed. Characterization of *Tpi* gene revealed abundance of TpiCa1a followed by TpiCa2b and TpiR1a strains respectively. The FAW population showed higher susceptibility towards chlorantraniliprole and spinetoram than cypermethrin. Insecticide resistance genes depicted marked upregulation although with lot of variance. Chlorantraniliprole resistance ratio (RR) exhibited significant correlation with 1950 (Glutathione S-transferase, GST), 9131 (Cytochrome P450, CYP) and 9360 (CYP) genes, while spinetoram and cypermethrin RR was found to correlate with 1950 (GST) and 9360 (CYP) genes.

**Conclusion:**

This study manifests Indian subcontinent as the potential new hotspot for the growth and distribution of FAW population that can be effectively controlled using chlorantraniliprole and spinetoram. This study also adds novel significant information on FAW population across Eastern India for developing a comprehensive pest management approach for *S. frugiperda*.

**Supplementary Information:**

The online version contains supplementary material available at 10.1186/s12896-023-00786-6.

## Introduction

Fall Armyworm (*Spodoptera frugiperda*, J.E Smith, Lepidoptera: Noctuidae) is a polyphagous pest natively growing in tropical and sub-tropical America by feeding on 353 plants from 76 plant families [1]. Its first occurrence report as an alien invasive pest traces back to Africa in 2016 [2]. Since then, the pest has rapidly distributed itself to other global regions causing significant damage to maize production [2–4]. Around mid-2018, FAW was first reported in the Indian subcontinent and majorly identified as maize feeders across the country [5]. However, within a short span it has rapidly spread to most of the regions in India and found to feed on crops other than maize including rice, sugarcane, sorghum and millets [6–8]. The reasons behind such alarming dispersal can be attributed to fast migration capacity, higher reproductive potential and polyphagous nature of the pest [9, 10]. Hence, developing a deeper understanding regarding the genetic background of FAW in the currently invaded Indian is the focus of the hour.

These FAW populations across the globe are divided into two phenotypically similar and genetically diverse subpopulations or strains, the rice-strain (RS) and the corn-strain (CS). These strains have been found to vary in their host plant selection, wing size and sex pheromone composition [11–13]. For DNA bar coding and strain confirmation (C or R) of FAW, the mitochondrial *cytochrome oxidase subunit I* (*COI*) gene sequence is most commonly used among many molecular markers, although ascertaining their host plant preference has not always been consistent using Mt*COI* gene [14]. For instance, some cornfield-collected populations of FAW were found to be the R-strain. In addition, Nagoshi et al. [15, 16] reported a nuclear *Triosephosphate isomerase* (*Tpi*) gene linked with Z-chromosome as another novel genetic marker. They stated in their work that majority of the corn field populations are hybrid (*Tpi*-C/*COI*-R) in nature, i.e., they harboured both a nuclear *Tpi*-C marker along with a mitochondrial *COI*-R marker. Hence, it was proposed that the host plant preference of the hybrid strain is associated with the nuclear *Tpi* marker, and therefore, Tpi gene is strongly hypothesized to be a more appropriate molecular marker for the identification of the genetic characteristics of FAW species related to the host plant choice [4].

The polyphagous nature of FAW along with its ability for evolution of resistance to chemical pesticides clearly reflects its strong environmental adaptability [17–21]. To mitigate the losses caused by this notorious pest, chemical control has always been considered as an effective strategy. Nonetheless, excessive reliance on synthetic insecticides has not only resulted in environmental damage but also led to resistance-development in the pest populations [22, 23]. Back in 1991, first insecticide resistance in *S. frugiperda* was noted for the carbaryl insecticide [24]. With the passing years, resistance built-up in FAW population to pyrethroids, organophosphates and diamide insecticides have also been reported [25].

The molecular mechanism underlying insecticide resistance include metabolic detoxification as one of the basic principles, primarily due to the activity of chief detoxifying enzymes, viz. cytochrome P-450 s (P-450 s), glutathione S-transferases (GSTs), etc. [26–28]. Reports have depicted that gene families regulating detoxification and metabolic events in FAW have widened [29, 30]. Interestingly, recent evidence indicates that Organophosphates (OP) and pyrethroid resistance in FAW is largely based on higher expression of different expressed sequence tags (ESTs) encoding GSTs, P450s and CEs enzymes. Among them GST 801, GST 968, EST 9555 and CYP 1950 are constitutively over-expressed in both OP and pyrethroid- resistant strains of FAW in USA. The mRNA levels of these genes are reported for its strong correlation with insecticide resistance in other insects [23].

There are no reports on the insecticide resistance mechanism of invaded FAW in India to date. Research concerning insecticide detoxifying genes (cytochrome P450s, GSTs) in FAW has largely been conducted by laboratory population. There exists few scarce studies on the genetic diversity of FAW in India since 2018, its first detection in India. Keeping in mind the prevalence of maize cultivation in the Eastern region of India, greater dependence on insecticides, wide dispersal capacity and broad-spectrum crop preference of this invasive pest FAW, it is crucial to experiment and understand the genetic diversity of the newly invaded FAW population along with the insecticide susceptibility status.

Hence, the aim of the present study is to evaluate the genetic diversity of FAW specimens collected from multiple locations around East India majorly on the basis of *COI* gene and delineate their disparity with respect to the current scenario of other geographical regions of the world. The second objective revolves around the evaluation of the insecticide-resistance risk among the experimental FAW population based on potential resistance-related genes. This study aiming to genetically define FAW population and comprehend their insecticide-resistance level will certainly expedite an enriched future pest-management for a secure agricultural practice globally.

## Results

### Strain-identification of FAW based on specific *COI* and *Tpi* gene

**Fig. 1:**
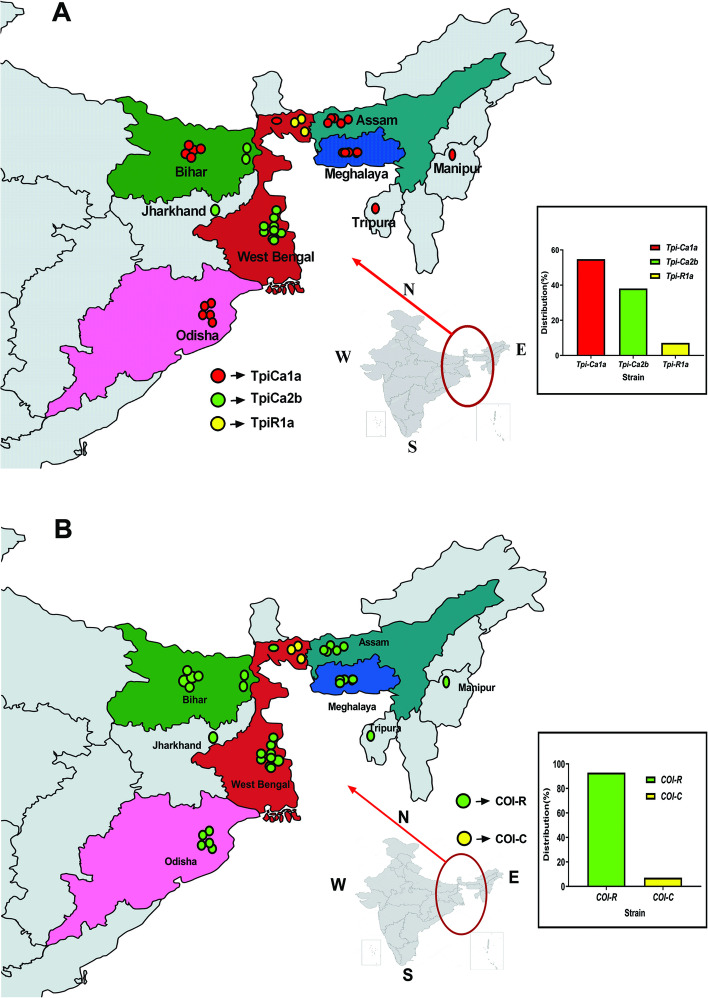
Map showing the collection sites of *Spodoptera frugiperda* specimens from the different provinces of Eastern India. Inset showing the complete map of India along with the collection sites of eastern India zoomed in and labelled accordingly (map has been taken from Barman et al., 2022 and modified for pictorial representation). **A** Inset shows the percentage distribution of collected strain of *Spodoptera frugiperda* based on *Tpi* gene. **B** Inset shows the percentage distribution of collected strain of *Spodoptera frugiperda* based on *COI* gene

In the present study, 42 FAW specimens collected from eight different provinces across Eastern India (as shown in Fig. 1) during 2019–2020 were subjected to molecular identification on the basis of *COI* marker gene sequence similarity. The generated *COI* sequences (as listed in Table S1) showed 100% similarity with *S. frugiperda* and were deposited in NCBI GenBank database. Furthermore, all the deposited *COI* sequences were analysed for in-depth study of population diversity across the regions. Few additional sequences of FAW were retrieved from African (*n* = 149), American (*n* = 164), and other Asian countries (*n* = 76) [i.e., Japan, Vietnam, Bangladesh, Myanmar, China, Pakistan, Korea] and were undertaken for comparative sequence analysis and inter-population variation. The details of these sequences are provided in Table S2.

**Fig. 2:**
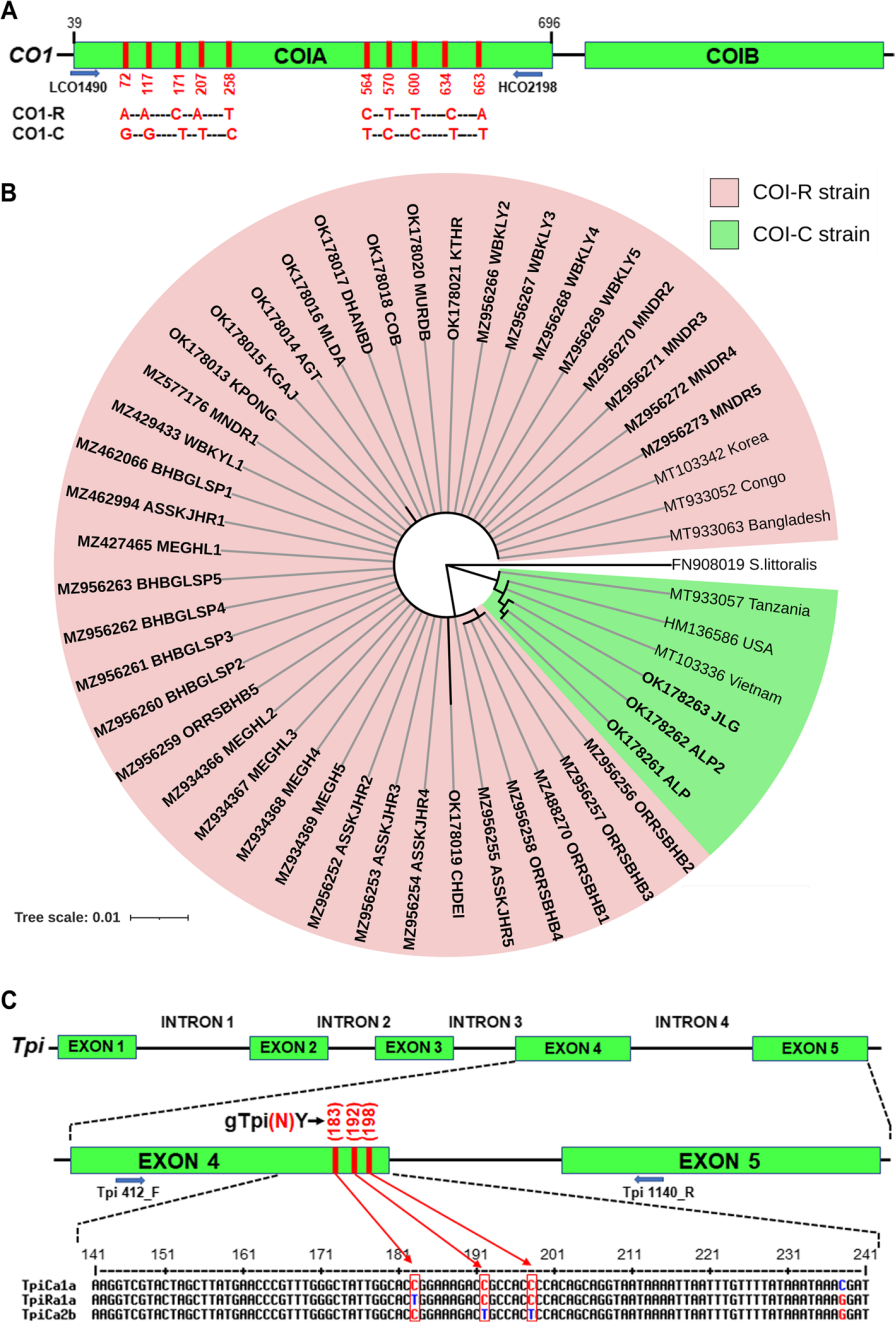
Strain identity and phylogenetic analysis of *Spodoptera frugiperda* based on *COIA* and *Tpi* gene segments. **A*** COI* gene showing the locations of *COIA* segment frequently used for DNA barcoding and individual nucleotide differences of the *COI* gene in the corn (*C*) and the rice (*R*) strains of *Spodoptera frugiperda*. 658 bp from 39 to 696^th^ positions of 1531 bp of *Spodoptera frugiperda COI* gene sequence has been used in this study. **B** The maximum likelihood phylogenetic tree of the *COI* sequences of *Spodoptera frugiperda* collected from the different provinces of Eastern India. The bold text *COI* sequences represent collected samples used in this study, and the others are reference sequences obtained from the GenBank database. A total of 49 nucleotide sequences were selected to construct the tree, where the *Spodoptera littoralis* was taken as an out-group. Hasegawa-Kishnio-Yano HKY850 model and gamma distribution rate of variation among sites were implemented to construct the phylogenetic tree in MEGA X. **C** Map of a section of the Z-chromosome linked nuclear *Tpi* gene that includes exon 4 (fourth exon from the start of the coding region), exon 5 and the intervening introns. The gTpi183Y site defines Tpi-based strain identity of FAW population indicated by C, T, C/T heterozygote forms. The Tpie4i4 contains a portion of exon 4 and intron 4

The existing strains of the collected population were distinguished by polymorphic sites in *COI* and *Tpi* genes alignments (Fig. 2A) [4]. Upon investigation, out of 42 COIA sequences from the studied area, 92.85% (39 sequences) of the samples belonged to ‘R’ strain, while the rest of the 3 specimens belonged to ‘C’ strain (Fig. 1B inset). Notably, the observations were consistent with the phylogenetic comparisons, the collected population majorly clustered as rice-strain (COI-RS) and the three samples (OK178262, OK178261 and OK178263) clustered as corn-strains (COI-CS) (Fig. 2B). Nonetheless, the discordance between the *COI* gene and FAW host association is apparent based on the fact that populations marked as rice-strain using *COI* marker were mainly collected from cornfields.

To understand the noted conflict, the nuclear *Tpi* gene marker was analysed, where strain identification is defined by the gTpi183Y polymorphism in *Tpi*-E4 region. The partial nucleotide sequence of *Tpi* gene, including 166 bp of *Tpi*-E4 region and 278 bp of *Tpi*-I4 region, aided in identification of three different strains across studied populations (*n* = 42), i.e., TpiCa1a (*n* = 23), TpiCa2b (*n* = 16), and TpiRa1a (*n* = 3) (Table S1). Results showed higher frequency of TpiCa1a strain (54.76%) followed by TpiCa2b (38.09%) and TpiRa1a (7.14%) (Fig. 1A inset). The genotype of each specimen was assigned based on single nucleotide polymorphism (SNP) between *Tpi*-E4 and *Tpi*-I4 regions. In the *Tpi*-E4 region, the gTpi183 loci of most of the samples (39 out of 42) were C indicating the dominance of corn strain in this region (Fig. 2C). The current study reports the prevalence of *Tpi*-R strain in Bengal for the first time, which was previously recorded from the states of Tripura and Karnataka [25]. A list of all *Tpi* gene sequences used in this study is provided in Table S1.

### Haplotype network and polymorphism analysis for FAW populations in India

**Fig. 3:**
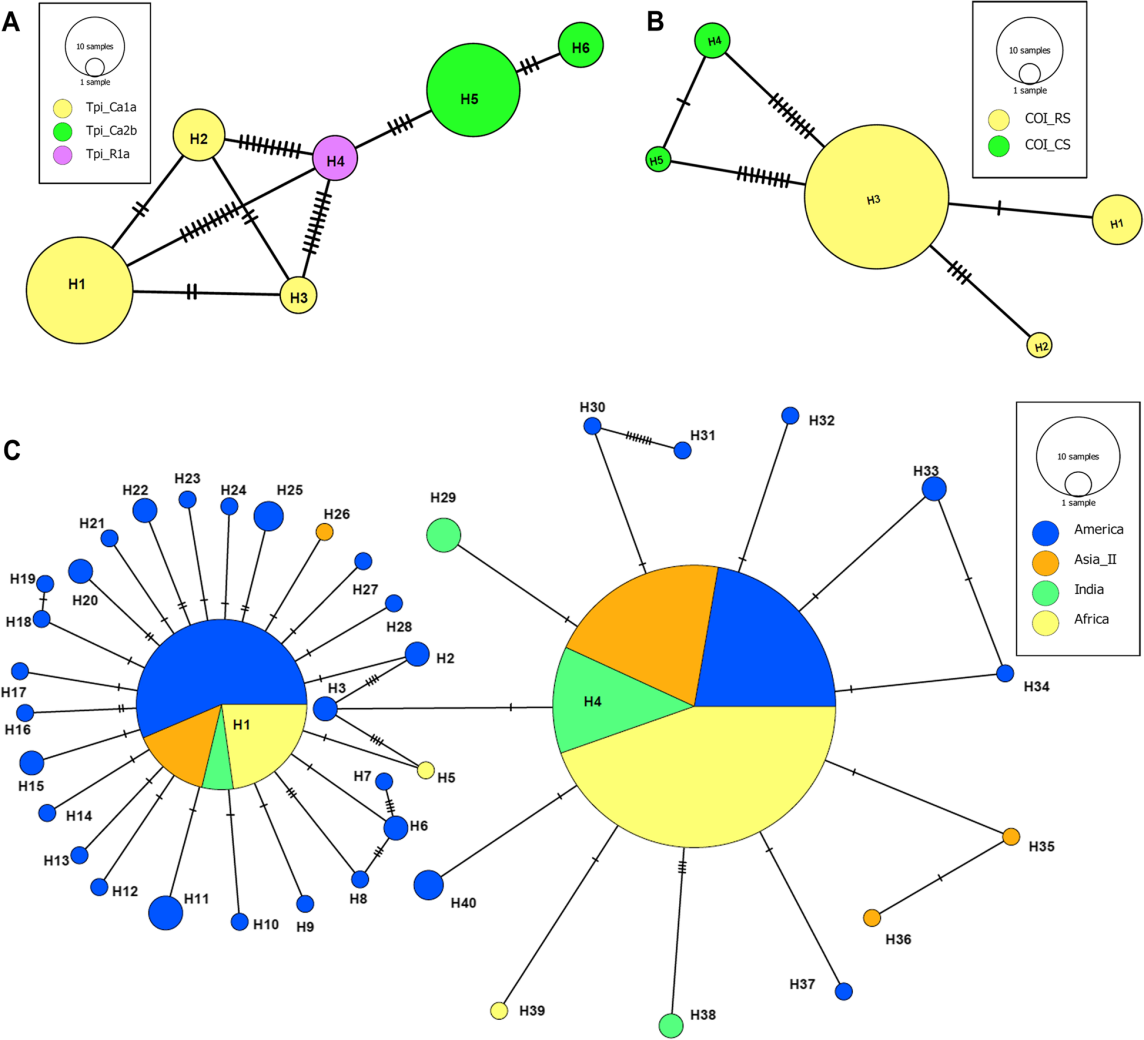
Polymorphism analysis of FAW population. Minimum spanning network analysis of *Tpi* gene (**A**) and the *COI* gene (**B**) haplotypes of *Spodoptera frugiperda* from the collected population. The three different strains Tpi-Ca1a (H1, H2 and H3), Tpi-Ra1a (H4) and Tpi-Ca2b (H5 and H6) were diversified into six haplotypes. **C** Haplotype network of partial mt-*COIA* gene sequences of FAW from four geographical group’s viz., India, Africa, America and Asia-II (labelled with separately coloured circles, as shown in the legend alongside). Each circle represents a unique haplotype, the frequency of which is proportional to the diameter of the circle. The hatch mark in the network represents the number of mutations separating the haplotypes from one another

The evolutionary relationship of the *Tpi* and *COI* gene was compared among the collected FAW population using minimum spanning network analysis (Fig. 3). The three different strains Tpi-Ca1a (H1, H2 and H3), Tpi-Ra1a (H4) and Tpi-Ca2b (H5 and H6) were diversified into six haplotypes that were quite distant from one another. Among the different haplotypes, Tpi-Ra1a (H4) occupied the central position of the network and was the linking haplotype between Tpi-Ca1a and Tpi-Ca2b with minimum mutational difference of 10 and 4 respectively (Fig. 3A).

On the other hand, only five haplotypes (H1 to H5) were identified based on the *COI* gene from the collected samples. Out of them three haplotypes (H1, H2 and H3) belonged to COI-RS and two (H4 and H5) belonged to COI-CS (Fig. 3B). Among the five different haplotypes, H3 occupied the central position of the network with a minimum mutational difference of 9 with both the *COI* corn strain (H4 and H5) (Fig. 3B).

For detailed understanding of the haplotype diversity of FAW, 460 bp of COIA barcode region for 42 specimens collected from Eastern India was analysed. 14 polymorphic sites (15 mutations) and a nucleotide diversity of 0.0075 were observed as shown in Table S3. Five different haplotypes from the studied COIA sequences with a haplotype diversity of 0.444 was identified, out of which three haplotypes belonged to the rice strain while the remaining belonged to corn strain. The number of polymorphic sites were higher in R strains (5 polymorphic sites and 8 singleton variable sites) as compared to the C strains (1 polymorphic sites and 8 singleton variable sites) whereas, nucleotide diversity was higher in C strain (0.0416) than in R strains (0.0071) (Table S4). Results obtained from the neutrality test (Fu and Li’s D*, Fu and Li’s F* and Tajima’s D) recorded a significantly negative value, indicating that the FAW population across this region might be undergoing expansion.

### Comparative genetic analysis across different geographical regions

Studying Indian FAW genetic variation in contrast to other global FAW populations is an intriguing aspect of the current study. This will help uncover the demography of FAW and the relationships among subpopulations. The global FAW population was split into four different groups wherein population-level studies were feasible. These groups included 1. America (containing population from North and South America); 2. Africa, 3. India (considering the population under study) and 4. Asia-II (including populations from Bangladesh, China, Korea, Vietnam, Japan, Myanmar and Pakistan). Sequences obtained from these particular groups were used for polymorphism study (Table S2).

The study of total 431 sequences revealed the prevalence of 41 haplotypes, among which American sub group had the highest number (35) of haplotypes. On the other hand, Indian and Asia-II subgroups had only 5 haplotypes, and African sub group consisted of 4 haplotypes. The number of polymorphic sites was higher in American population (46 polymorphic sites and 8 singleton variable sites) followed by Indian (14 polymorphic sites and 8 singleton variable sites) and Asia-II population (12 polymorphic sites and 8 singleton variable sites). As shown in Table S3, haplotype diversity was also higher in American population (0.746) as compared to Indian (0.444), Asia II (0.383) and African population (0.285); whereas, nucleotide diversity was higher in Indian (0.0075) followed by African (0.0018), American (0.0006) and Asia-II (0.0003) populations. From the above statistics, it can be confirmed that population in India and the ones outside India are still in a state of expansion (owing to large Hd value and small π). This statement is further supported by the Minimum spanning network, a characteristic star shaped network observed during population expansion [24] (Fig. 3C). There appears to be a significant interaction among the FAW population of different geographical ranges despite the thousands of kilometres that separate each of these locations.

**Fig. 4:**
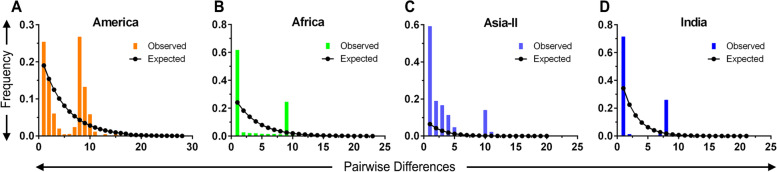
Mismatch distribution plot of FAW population. Mismatch distribution analysis of the *Spodoptera frugiperda* mt-DNA combined dataset from four geographical group’s viz., America (**A**), Africa (**B**), Asia-II (**C**) and India (**D**). The vertical axis represents the frequency, whereas the horizontal axis shows the number of pairwise differences. The dotted line represents the expected distributions for a population, and the bar chart represents the observed distribution for each

Mismatch distributions analysis conducted to understand further demographic processes revealed distributions were distinctly unimodal for the four groups under study (Fig. 4). This is indicative of the population undergoing a bottleneck in population size followed by rapid expansion [24]. A very recent introduction of FAW into India justifies the occurrence of rapid expansion. The findings of neutrality test also corroborated with the above statement. However, maximum genetic variation was observed in American population (Fig. 4A) as opposed to Indian population (Fig. 4D) and it is likely to be possible that a large amount of genetic diversity is lost in the course of adaptation to newer habitats [31]. Also, the results reflect the recent invasion of FAW in the other two sub-groups, Africa and Asia II (Fig. 4B and C).

### Genetic segregation within different population

The present study also focused on the population structure of four geographical groups using AMOVA. Primarily the four geographical groups were compared and the same analysis was individually performed between India and America; India and Africa; India and Asia-II populations (Table S5). The results revealed significant genetic discrimination between the four different groups (15%) indicating the existence of genetic structuring between them. Hierarchical AMOVA revealed that African and Asia-II population has lowest genetic differentiation with respect to the invasive Indian population (9% and 29% respectively) contrary to American population where maximum genetic variation (69%) was observed. As depicted from Table S5, a low genetic variation and F_ST_ value indicate genetic homogeneity and lack of genetic structuring between the population of India and Africa.

### Susceptibility of FAW to three insecticides

**Fig. 5:**
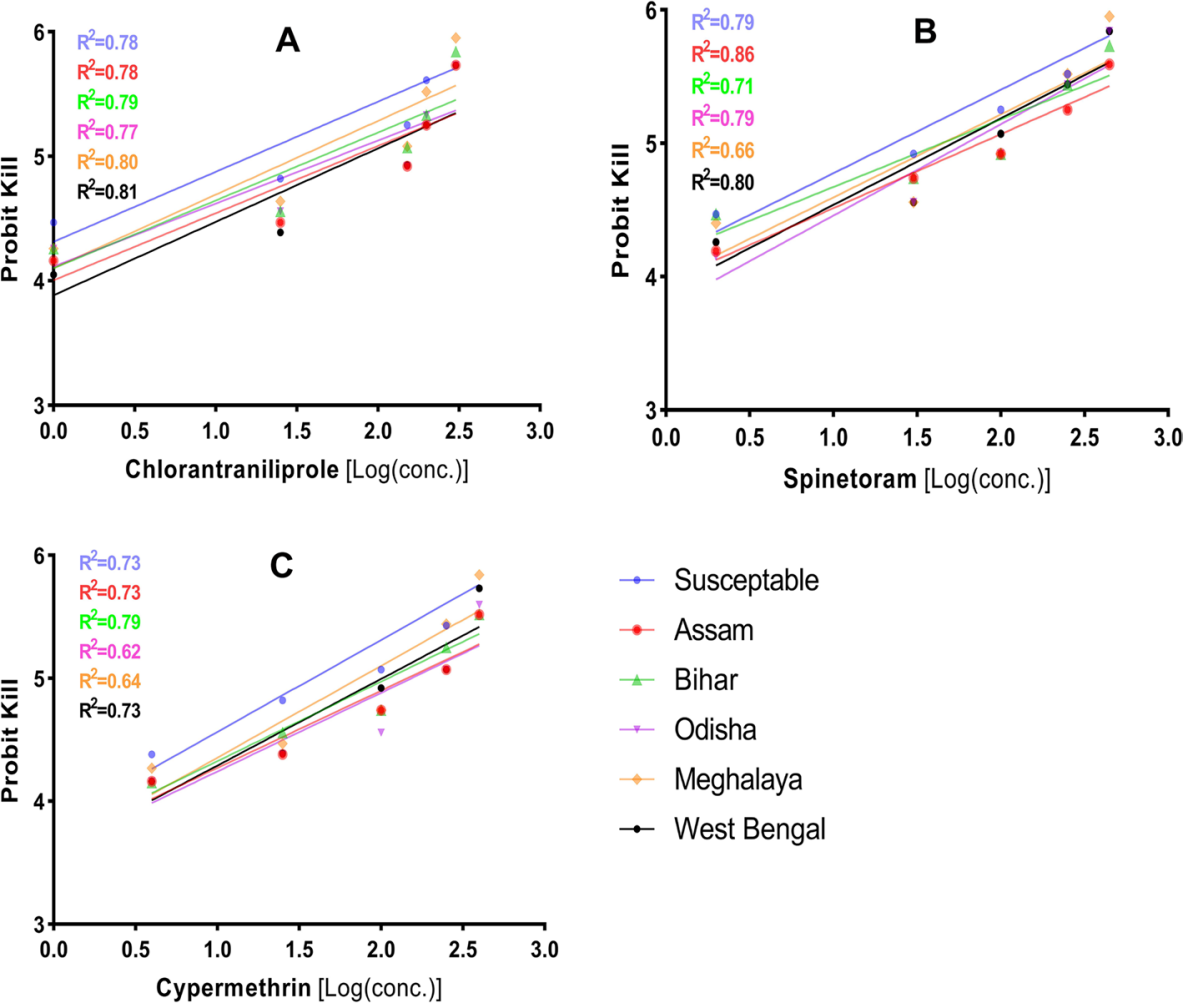
Mortality response of FAW population to insecticides. Increasing concentrations of three insecticides, chlorantraniliprole (**A**), spinetoram (**B**) and cypermethrin (**C**) was tested against different populations of FAW labelled with separately coloured lines, as marked in the legend) to analyse the mortality response. X-axis represents the logarithmic dose of every insecticide concentration tested, while Y-axis represents the probit kill values. The straight line represents the best fitting probit regression line between insecticide log dose and mortality probits (working probits) for FAW Larvae. The R^2^ of each individual line in the graphs represent regression coefficient. Slopes of the lines are given in Table S6

The susceptibility of different populations of FAW was evaluated against three insecticides viz*.,* chlorantraniliprole, spinetoram and cypermethrin (Fig. 5). The results as shown in Table S6 indicate that the toxicity (LC_50_) level varies between 17.60–89.05 mg/L for chlorantraniliprole (Fig. 5A), 23.87–131.28 mg/L for spinetoram (Fig. 5B) and 39.98–149.41 mg/L for cypermethrin (Fig. 5C), signifying higher susceptibility of the population towards these novel group of insecticides (chlorantraniliprole and spinetoram). Based on the LC_50_ value of the susceptible laboratory population, chlorantraniliprole (LC_50_ = 17.60 mg/L) was found to be the most toxic followed by spinetoram (23.87 mg/L) and cypermethrin (39.98 mg/L). The populations belonging to Assam, Bihar, Meghalaya and Odisha followed a similar toxicity trend for these insecticides. However, the FAW population collected from West Bengal showed higher toxicity towards spinetoram (LC_50_ = 53.45 mg/L) followed by chlorantraniliprole (89.05 mg/L) and cypermethrin (105.75 mg/L).

The insecticide susceptibility comparison between the different populations indicated that the resistance factor (RF value) varies between 1.97–5.06, 1.95–5.50 and 1.93–3.74 for chlorantraniliprole, spinetoram and cypermethrin, respectively (Table S6). The maximum RF value for chlorantraniliprole was recorded in the population of West Bengal (5.06) where the lowest (1 ppm) and highest (300 ppm) tested concentration resulted in working probit kill of 4.05 and 5.73. Toxicity comparison with the laboratory population for spinetoram indicated the highest RF value (5.50) for Assam population (Table S6). Assam population showed working probit kill value of 4.19 and 5.59 for lowest (2 ppm) and highest (450 ppm) test concentration, respectively. In case of cypermethrin, the maximum RF value (3.74) was observed in Odisha population (Table S6), wherein the lowest (4 ppm) and highest (400 ppm) tested concentration resulted in working probit kill of 4.16 and 5.60.

### Expression of resistance genes correlated with resistance ratio of the insecticides

**Fig. 6:**
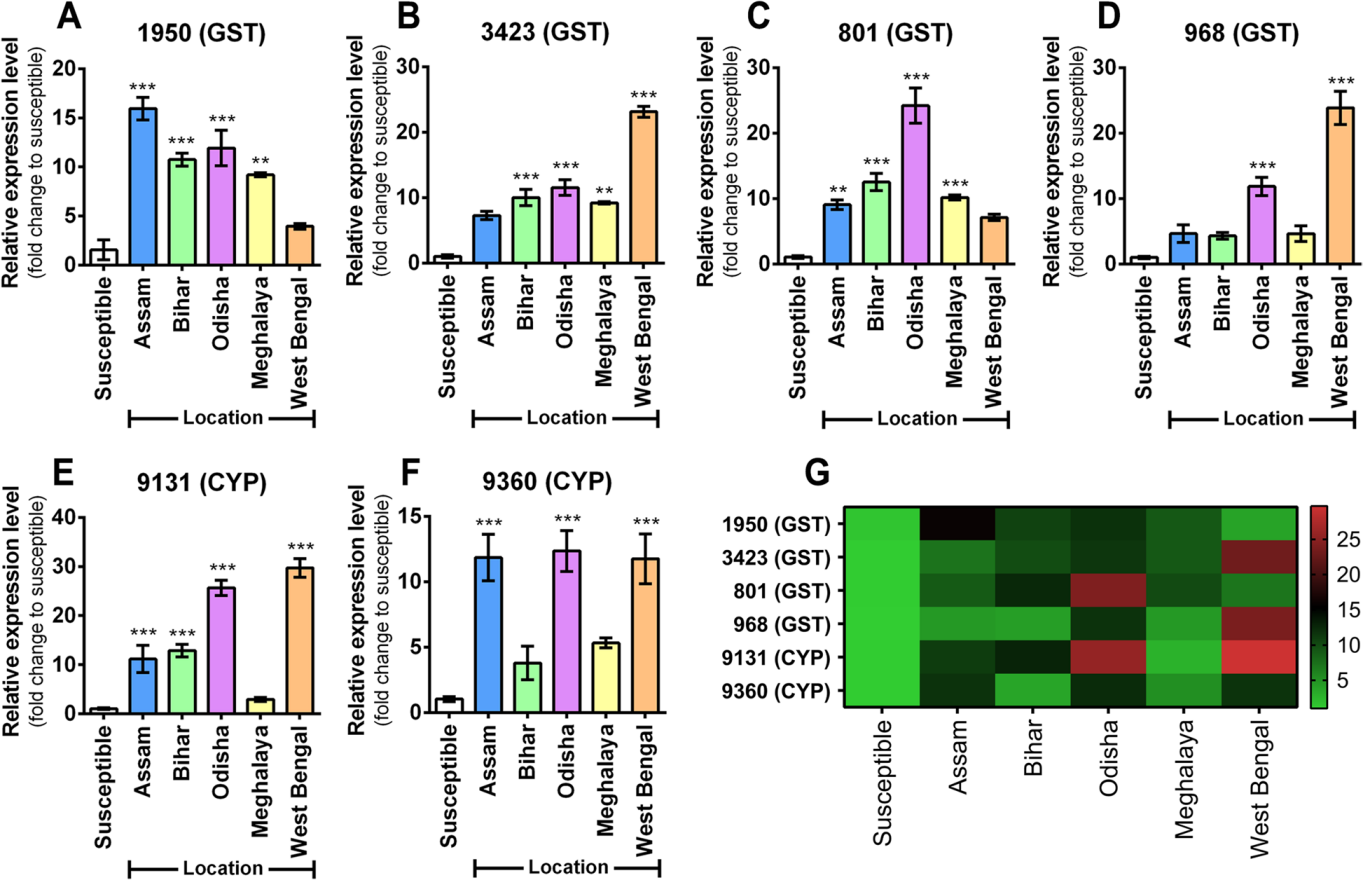
Expression analysis of insecticide resistance genes in FAW population. Expression profiles of six insecticide resistance genes (4 GST and 2 CYP 450) in FAW populations (as listed along the X-axes). **A** 1950 (GST), **B** 3423 (GST), **C** 801 (GST), **D** 968 (GST), **E** 9131(CYP) and **F** 9360(CYP). Relative gene expression was measured by qRT-PCR analysis. Elongation factor (EF) has been used as the internal control. The Ct value for tested genes were normalized to the Ct value of EF and calculated relative to a calibrator using the formula 2^–ΔΔCt^. Values represent means ± SE for three independent replicates. Statistical significance of the experimental sets as compared to the susceptible population have been marked with ***p ≤ 0.0001. **, *p* ≤ 0.001 and * *p* < 0.01. **G** Heatmap represents an overview of the expression level of each gene in variable FAW population, with the color gradient provided alongside. Gene expression profile are represented as relative expression as compared to susceptible population, obtained from qRT-PCR experiment

Expression of six EST genes (four GST genes and two P450 genes) was compared in 5 FAW populations against a susceptible reference population (Moundouri, West Bengal). As compared to the susceptible population, all the experimental FAW populations displayed upregulation of all the six genes, although at varying degrees. In case of 1950 (GST), Assam, Odisha and Bihar populations showed maximum upregulation (11–16-fold) followed by Meghalaya and West Bengal populations depicting ninefold and fourfold upregulation respectively (Fig. 6A and G). Next, for 3423 (GST) and 968 (GST) transcript level, West Bengal population exhibited the highest upregulation (23-fold) followed by Odisha and the other populations with 12-fold and 4–tenfold expression upregulation respectively. (Fig. 6B, D and G). Relative expression of 801 (GST) was moderately high in all the populations (7–12-fold) with maximum expression in Odisha population (24-fold) (Fig. 6C and G). Relative expression of 9131 (CYP) gene was maximum in West Bengal (30-fold) and Odisha populations (26-fold), moderately high in Bihar and Assam populations (11–13-fold) and very minute upregulation in Meghalaya population (Fig. 6E and G). Lastly, for 9360 (CYP) transcript level, maximum upregulation was noted in Odisha, West Bengal and Assam population (around 12-fold) while Meghalaya and Bihar population showed less upregulation (3–fivefold) (Fig. 6F and G). It shows that West Bengal and Odisha population harboured maximally upregulated insecticide resistance genes majorly while Meghalaya population is comparatively more susceptible than others.

**Fig. 7:**
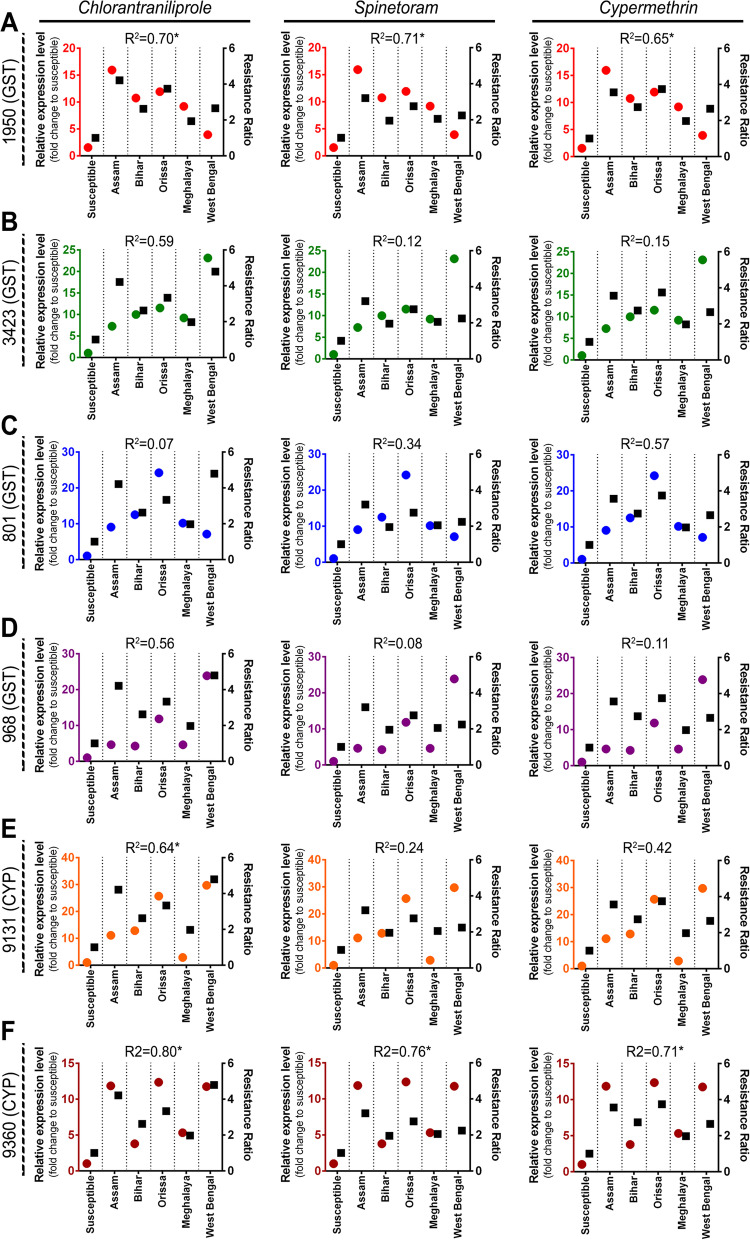
Linear regression analysis between insecticides resistance level and relative expression of insecticide resistance genes. Linear regression analysis was used to test for functional relationship between the resistance level of each insecticide (chlorantraniliprole, spinetoram and cypermethrin) and the mean normalized expression value of each insecticide resistance gene in different FAW population, where **A** 1950 (GST), **B** 3423 (GST), **C** 801 (GST), **D** 968 (GST), **E** 9131(CYP) and **F** 9360(CYP). Circles in each graph represent the gene expression values (marked in left y-axis) while squares depict the resistance ratio of each insecticide (labelled in right y-axis)

The correlation coefficient between resistant ratio (RR) values of FAW field population and relative expression of detoxifying genes is presented in Fig. 7. The expression levels of 1950 (GST), 9131 (CYP), 9360 (CYP) were significantly correlated with resistance ratio (RR) of chlorantraniliprole. The correlation coefficients were 0.70 (*p* = 0.03), 0.64 (*p* = 0.05) and 0.80 (*p* = 0.01) respectively. Similarly, both the resistant ratio of spinetoram and cypermethrin exhibited a positive correlation with the transcript level of 1950 (GST) and 9360 (CYP). The correlation coefficient was 0.71 (*p* = 0.03) and 0.76 (*p* = 0.02) for spinetoram while, 0.65 (*p* = 0.05) and 0.71 (*p* = 0.03) for cypermethrin respectively (Fig. 7).

## Discussion

Fall armyworm is considered as one of the highly invasive and potentially threatening insect pests of agricultural crops. The rapid evolution and spread of FAW has garnered huge attention worldwide. Therefore, precise assessment of its genetic identity (strain and pesticide resistance properties) is a pre-requisite for risk assessment and development of pest-management strategies. Tracing the evolutionary course of any population over geography is ideal for population genomics study. However, very few studies analysing the genetic diversity of Indian FAW population have been reported and there seems to be a substantial lacuna in this field of research [25]. To get a clear insight regarding the genetic background of the invasive insect species, 42 specimens of FAW across different regions in Eastern India were characterized using molecular markers of both *Tpi* and *COI* genes. This study also observed a disagreement between *Tpi* and *CO1* gene markers for the estimation of host association. Varying proportions of different genetic groups were seen using markers (*Tpi* and *CO1* gene) individually. However, this observation aligns well with the reports from other regions of the world [4].

Results showed that most of the FAW specimens under study had *Tpi*-C genotypes, however, *COI* gene analysis revealed more than 90% of the population to belong to *COI*-R genotype. The incongruity between the *COI* and *Tpi* gene marker for prediction of host association is probably owing to the fact that most of the specimens having *COI*-R genotype were mainly collected from cornfields. Nevertheless, three of the collected specimens belonging to *Tpi ‘R’* strains were also collected from corn fields. So, it is yet to be confirmed whether the unique TpiRa1a specimens in India showed a preference towards rice strain hosts or not. Although, there are extensive rice planting areas in Eastern India, no reports are indicative of serious damage to rice caused by FAW in this region. Furthermore, by comparing the results of *COI* and *Tpi* genes, it is apparent that most of the samples being studied were predominantly hybrid (*Tpi*-C/*COI*-R). A recent study indicated that most of the Indian strains were particularly hybrid (*Tpi*-C/*COI*-R) as marked by the discordance between *Tpi* and *COI* markers [25]. Thus, investigating the nuclear *Tpi* gene is a better alternative to predict host plant association unambiguously [4]. Our study also aimed to investigate the genetic variation of *COI* gene in FAW among different groups where invasion of this species has taken place recently. As reported earlier, genetic variability helps in inferring the demographic history of a population [32]. Results from the analysis of mt*COI* sequences indicate genetic homogeneity among the African and Asian population. It is also evident that population in India as well as outside India is in a state of expansion (large HD and small π). A low genetic variation among the geographical groups (India vs Africa and India vs Asia II) were observed as represented in the AMOVA (Table S5) and the low F_**ST**_ value between them validates the observations on genetic homogeneity and lack of genetic structuring.

The finding from the current study may be endorsed by the influences of ecological as well as physiological suitability of the FAW in Indian subcontinent, as well as its potential to fly and establish itself in new environment [9]. Prevalence of two major strains (referred to as C-strain and R-strain) not only results in different host plant preference but also diverse dispersal pattern and differential response to pesticides [26, 27]. Considering the rampant distribution of rice and corn haplotypes throughout the Indian subcontinent along with prevalence of rice and maize planting areas, migration of FAW across the country seems unrestrained [25]. Consequently, FAW population has a high chance of developing adaptive advantage in different regions of the country. Hence, it is only prudent to develop efficient management strategies to control the notorious pest. However, occurrence of different strains will make the task complex due to their differential responses to insecticides. Moreover, rapid evolution of insecticide resistance is of grave concern to the farmers and scientists across India. Several global studies have highlighted the resistance development in different population of FAW to different groups of insecticides [28]. However, literature focusing on the susceptibility status of FAW population to insecticides in Eastern India is limited. In this regard, field population of FAW surveyed from different locations in Eastern India were tested for their susceptibilities to three commonly used insecticides in these regions. Results revealed that among the three test insecticides (chlorantraniliprole, spinetoram and cypermethrin), moderate to high susceptibility of the populations was recorded towards the two novel insecticides (chlorantraniliprole and spinetoram).

Underlying reasons for resistance development against pyrethroids in FAW have been widely attributed to insensitivity of the target site and detoxification of insecticides by metabolic enzymes [29]. A study on the genetics of resistance in *S. frugiperda* to a commonly used pyrethroid (lambda-cyhalothrin) has indicated involvement of multiple recessive genes [30]. Reports of resistance to organophosphate (OP) and pyrethroid (PYR) has already surfaced in America and Africa [22, 33–35]. Since, comparative genetic analysis across different geographical regions signifies a genetic homogeneity and lack of genetic structuring between the population of India and Africa, there are ample chances for development of resistance in Indian population of FAW in the near future. Field experimental studies also suggested cypermethrin to be less efficient in controlling FAW as compared to other insecticides in India [36]. Although the bioassay study here also revealed cypermethrin to not have high toxic effects on FAW, it is still largely used to control FAW in India because of minimal risk it causes to humans and the environment [37].

Molecular mechanism underlying resistance involved enhancement of metabolic detoxification by microsomal oxidases (MFOs), GSTs, hydrolases and reductases resulting in FAW resistance to pyrethroids, organophosphates and carbamate group of insecticides [38]. Microarray studies also revealed that genes encoding GST, cytochrome P450s and carboxylesterases (CEs), were over-expressed in the OP and PYR-resistant strains of FAW [23]. Several GSTs, P450s and CEs, over expressed in the resistant strains which was seen to be strongly correlated with insecticide resistance in laboratory populations of FAW [39]. In the current study, a significant positive correlation was noted between the expression of 1950 (GST), 9131 (CYP) and 9360 (CYP) with resistant level in different field populations of FAW. The results are indicative of the fact that these detoxifying genes are upregulated not only in laboratory populations but also in field populations. It is quite plausible that multiple enzymatic interaction with varying expression pattern might be governing the differential susceptibility of FAW strains against different insecticides.

## Conclusion

This research article provides a genetic “snapshot” of FAW populations covering Eastern India describing the situation after two years of its invasion. Since FAW has began to broaden its host crop preference and continues to adapt to a new environment, this study would be useful to establish the connotation between host range and FAW haplotypes across the country and also help in evaluating the efficiency of different insecticides. Moreover, this study also indicates that Indian subcontinent might be developing as the new hotspot for FAW expansion of probably the rice COIA haplotypes. Results of bioassay indicate moderate to high susceptibility of FAW population towards selected insecticides. This is notably reflected in population genetics study which also reveals that invaded FAW is genetically homogenous owing to its recent invasion. As the population is genetically homogenous, it will not show a differential response to pest management strategies, which further aids in designing tools for management. However, the link between insecticide resistance level and the relative expression of different insecticide resistance genes signifies their possible involvement in development of FAW resistance in the near future. So, we hypothesized that the dynamics of FAW resistance towards different insecticides isn’t affected by genetic homogeneity, but rather by spatio-temporal gene expression (especially those associated with insecticide resistance). Such gene expression studies will also be a good basis for better management measures. On a concluding note, this study shall serve as a useful reference point for awareness regarding the susceptibility level of insect populations in different geographical regions and will play a vital role in evaluating the trends in spatial and temporal resistance development.

## Methods

### Insect collection, extraction of genomic DNA and PCR-based amplification

In 2019–20, 42 FAW specimens (larvae) were collected from 8 diverse provinces in Eastern regions of India, comprising of Assam, Bihar, Odisha, Meghalaya, West Bengal, Tripura, Manipur and Jharkhand as displayed in Fig. 1. The collection sites have been listed in Table S1. The lower abdomen portion of each specimen was used for the purpose of genomic DNA extraction as per manufacturer’s instruction using gDNA tissue kit (Invitrogen, USA), followed by an analysis via Qubit Flex Fluorometer (Thermo Fisher Scientific) and storage at − 80 °C until use.

On one hand, amplification of a partial sequence (444 bp) of the *Tpi* gene was carried out using the primer pair TPI412F and TPI1140R whereas, on the other hand, the partial sequence (658 bp) of the *COI* gene was amplified using LCO1490 and HCO2198 primer pair in Veriti 96-Well Thermal Cycler [Applied Biosystems, USA] [40]. Primer details are listed in Table S7. The amplified PCR products were purified using the XcelGen DNA Gel/ PCR Purification mini kit (Xcelris genomics, India) following manufacturer’s instruction followed by DNA sequencing by Sanger dideoxy method from Chromus Biotech Laboratory, India.

### Characterization of the *COI* and *Tpi* gene segments

Diagnostic markers to differentiate between strains were based on single nucleotide substitution of *Tpi* and *COI* genes [40]. The *Tpi* gene was termed as “g” (genomic), whereas the *COI* gene was labelled by “mt” (mitochondria, followed by gene name, base pairs number from the anticipated translational start site). In order to classify the C and R strains of FAW by marking the polymorphic nucleotides in *Tpi* and *COI* gene, the sequences of the collected FAW specimens were aligned with the earlier reported NCBI sequences of C-strain and R-strain FAW in CLUSTAL W. The alignments of the *COI* gene (mitochondrial) fragment having single nucleotide polymorphism (SNP) often nucleotides (mtCOI72, mtCOI117, mtCOI171, mtCOI207, mtCOI258, mtCOI564, mtCOI570, mtCOI600, mtCOI634, and mtCOI663) were strain-specific and used to indicate the difference between ‘R’ and ‘C’ sister strains of FAW in the Western Hemisphere [4]. Nagoshi et al. [4] too testified the C- and R-strains by means of several polymorphic nucleotides in the exon (*Tpi*-E4) and intron region (*Tpi*-I4) of the *Tpi* gene. While, the gTpi183 (C for C-strain, T for R-strain) helped to identify C- and R-strains [4], gTpi192 and gTpi198 served to classify subgroups (Tpi-Ca1, Tpi-Ca2, and Tpi-Ca1/Ca2) of C-strain. Moreover, numerous polymorphic nucleotides in *Tpi*-I4 aided in the understanding of genetic variation of FAW population.

### Genetic structuring and phylogenetic analysis

The demographic history of each geographical group of FAW was analysed by different neutrality statistics. Tajima’s D [41] and Fu’s Fs [42] statistics were consequently calculated to analyse whether *COI*A followed the expectations of neutrality by DnaSP. Mismatch distribution graphs for each geographic groups were created through DnaSP Ver. 5.10.01 [43] to infer if FAW underwent demographic expansion. The population pairwise F-statistics (F_ST_) and analysis of molecular variance (AMOVA) were calculated by Arlequin v.3.5 [44]. The sequence haplotype network was scrutinised using minimum spanning network relationship through popART software. Genomic DNA sequences of *Tpi* and *COI* genes were submitted to the GenBank (http://www.ncbi.nlm.nih.gov) with accession no. as detailed in Table S1. Evolution in *COI* gene sequences were studied via a phylogenetic tree construction using MEGA X software [45], verified by maximum likelihood (ML) model with 1000 bootstrap replicates. The iTol (version 6.4.3) was used for graphical representation of the phylogenetic tree.

### Insecticide susceptibility bioassay test

In order to evaluate the insecticide susceptibility and expression status of resistant genes, the field FAW populations from five most significant provinces (one from Assam, Bihar, Odisha, Meghalaya and West Bengal each) were chosen, wherein, FAW were identified on fields based on the larval morphological features [2]. The accumulated larval populations were continued in control condition (25 ± 1 °C, 70–80% RH, 16L: 8D photoperiod), reared on maize plants, the adult FAWs were fed with 10% sugar solution and maintained in cages (30 cm diameter × 45 cm height) internally covered with crocus cloth (oviposition substrate). The newly laid eggs were disinfected with 5% formaldehyde. The second instar larvae (5–6 mg) of F1 generation from each FAW population were used for the bioassays [46]. The FAW population collected from the university farm (B.C.K.V, Moundouri, India) were selected to be reared for ten further generations barred from any insecticide exposure to be considered as the susceptible population (SP) [47].

Insecticides viz., chlorantraniliprole 18.5% SC (Coragen) obtained from Du Pant Co., Ltd., cypermethrin 10% EC (Super killer) from Dhanuka Chemical Ltd. and spinetoram 11.7 SC (Delegate) from Dow Agro Sciences served as the test agent in bioassay studies of the FAW larval population. A modified leaf dip bioassay method of Insecticide Resistance Action Committee was followed [48]. For toxicity evaluation, final doses were finalised from the earlier laboratory population experiments. For every insecticide, fresh maize leaves (5 X 5 cm^2^) were dipped in the respective insecticide dilutions for 30 ± 2 s and air-dried. The control leaves were dipped in water. After complete drying of the solution on the leaf surface, the treated leaves were then shifted to 2% agar layer. Five replicates of each treatment were used and six larvae were released per replication (thirty in each treatment) in the experiment being performed under laboratory conditions (25 ± 1 °C, 70–80% RH, 16L: 8D photoperiod). Following every 24 h, the treated leaves were changed with freshly treated leaves. The mortality counts (moribund larvae were considered as dead) were noted at 96 h after feeding (HAF).

### Insecticide resistance genes expression analyses

The field-collected FAW (2^nd^ instar) population were used for total RNA extraction using Insect RNA Isolation Kit (Thermo Fisher Scientific) following the manufacturer's protocol, followed by amount estimation using Qubit Flex Fluorometer. 1 μg of total RNA was converted to cDNA according to manufacturer’s instruction (GeneSure H-Minus First Strand cDNA Synthesis Kit, Genetix Biotech Asia Pvt. Ltd.).

The qRT-PCR assay was conducted to analyse the expression pattern of resistance marker cytochrome P450 and GST genes namely, 9131 (CYP), 9360 (CYP), 1950 (GST), 3423 (GST), 801 (GST), 968 (GST) using SYBR Green Master Mix (Applied Biosystems, USA) in Agilent Technologies Stratagene (Model- Mx3000P). Primer details including name and sequences along with annealing temperatures have been enlisted in Table S7. 2^−ΔΔCt^ method [49] has been used to calculate the relative expression of each target genes with reference to the internal control EF (Elongation factor) gene.

### Statistical analysis

The logged mortality counts corrected using Abbott’s formula [50] were used to calculate the lethal concentration (LC) values. Probit analysis of the data was performed using SPSS [51]. The resistance ratio (RR) for each population was calculated by: LC_50_ of the test population/LC_50_ of the susceptible population [52], and then classified as follows: RR < 5 low resistance; RR = 5–10 moderate resistance; RR =  > 10 high resistance [53]. The qRT-PCR was conducted at least thrice with a minimum of three discrete biological replicates. Data is represented as means ± SD. One-way analysis of variance (ANOVA) followed by Dunnett's multiple comparison test was used for statistical significance determination in Graph Pad Prism 6.0, wherein significant differences with the susceptible population is represented as *p < 0.01, **p < 0.001, and ***p < 0.0001. The functional relationship between the resistance level of the population and the mean normalized expression value of each gene in different populations was determined by Linear Model II regression analysis in SPSS (SPSS for Windows, Rel. 17.0.0 2009. Chicago: SPSS Inc.).

## Supplementary Information

**Additional file 1: Table S1.** Collecting information of *Spodoptera frugiperda* from different prefectures in 2020. **Table S2.** COIA gene sequences across different geographical regionused in the present study. **Table S3.** Summary of genetic diversity of FAW populations analysed on the basis of partial mt-COIA gene from four different geographical location i.e., India, America, Africa and Asia-II.. **Table S4.** Comparison between genetic diversity of FAW sister strains in India.. **Table S5.** Result of AMOVA analysis among the different FAW geographical groups. **Table S6.** Susceptibility of *Spodoptera frugiperda* field populations to three insecticides. **Table S7.** Primers used in the current study. The primer name, PCR type, primer sequences and the annealing temperature are listed in the table below.

## Data Availability

The raw sequencing data were deposited in the NCBI (Gene Bank) repository under the accession number MZ427465, MZ462994, MZ488270, MZ462066, MZ429433, MZ577176, MZ934366, MZ934367, MZ934368, MZ934369, MZ956252 MZ956273, MZ956260-MZ956263, OK178013, OK178021, OK178261- OK178263, MZ579532- MZ579537, MZ971201- MZ971205, MZ971182- MZ971200, OK247557- OK247565, OK571339- OK571341.
