# Supplementary material for: Evidence of population expansion and insecticide resistance mechanism in invasive fall armyworm (Spodoptera frugiperda)
Source: BMC Biotechnol. 2023 Jul 4;23:17. doi: 10.1186/s12896-023-00786-6 (PMC10318647; doi:10.1186/s12896-023-00786-6)
Supplement: Supplementary file 1 — Additional file 1: Table S1. Collecting information of Spodoptera frugiperda from different prefectures in 2020. Table S2. COIA gene sequences across different geographical regionused in the present study. Table S3. Summary of genetic diversity of FAW populations analysed on the basis of partial mt-COIA gene from four different geographical location i.e., India, America, Africa and Asia-II.. Table S4. Comparison between genetic diversity of FAW sister strains in India.. Table S5. Result of AMOVA analysis among the different FAW geographical groups. Table S6. Susceptibility of Spodoptera frugiperda field populations to three insecticides. Table S7. Primers used in the current study. The primer name, PCR type, primer sequences and the annealing temperature are listed in the table below. [file 12896_2023_786_MOESM1_ESM.docx]

**Evidence of population expansion and insecticide resistance mechanism in invasive Fall Armyworm (*Spodoptera frugiperda*)**

Snigdha Samanta^1#^, Mritunjoy Barman^1,5^ ^[[1]](#footnote-1)^#, Himanshu Thakur^2^, Swati Chakraborty^3^, Gouranga Upadhyaya^4^, Deepayan Roy^5^, Amitava Banerjee^1^, Arunava Samanta^1^, Jayanta Tarafdar ^3^*

^1^Department of Agricultural Entomology, Bidhan Chandra Krishi Viswavidyalaya, Mohanpur, West Bengal, India.

^2^Department of Entomology, C.S.K. Himachal Pradesh Krishi Vishvavidyalaya, Palampur, Himachal Pradesh, India

^3^Department of Plant Pathology, Bidhan Chandra Krishi Viswavidyalaya, Mohanpur, West Bengal, India.

^4^Department of Biological Sciences, Indian Institute of Science Education and Research Kolkata, West Bengal 741246.

^5^School of Agriculture Science, GD Goenka University, Gurugram, Haryana 122103

^*^Author for correspondence:

Email: jayanta94bckv@gmail.com; Telephone: +919830342320

**Table S1**: Collecting information of *Spodoptera frugiperda* from different prefectures in 2020.

| **Sl.**  **No.** | **GenBank Accession** | | | | **Collection sites** | **Type of experiment** | | **Location coordinates** | **Country** |
| --- | --- | --- | --- | --- | --- | --- | --- | --- | --- |
|  | **COIA**  Strain- CO1-R/CO1-C | | **Tpi**  Strain- TpiCa1a/TpiCa2b/Tpi-R | |  |  |  |  |  |
| **1** | MZ427465 | R | MZ579533 | TpiCa1a | Tura, Meghalaya | Bioassy/ Population genetics | | 25.51°N, 90.20° E | India |
| **2** | MZ462994 | R | MZ579535 | TpiCa1a | Kokrajhar, Assam | Bioassy/ Population genetics | | 26.40°N, 90.26 ° E | India |
| **3** | MZ488270 | R | MZ579536 | TpiCa1a | Bhubaneswar, Odisha | Bioassy/ Population genetics | | 20.29°N, 85.82 ° E | India |
| **4** | MZ462066 | R | MZ579534 | TpiCa1a | Bhagolpur, Bihar | Bioassy/ Population genetics | | 25.24°N, 86.98° E | India |
| **5** | MZ429433 | R | MZ579532 | TpiCa2b | Kalyani, West Bengal | Bioassy/ Population genetics | | 22.97°N, 88.43° E | India |
| **6** | MZ577176 | R | MZ579537 | TpiCa2b | Moundouri, West Bengal | Bioassy/ Population genetics | | 21.83°N, 87.42° E | India |
| **7** | MZ934366 | R | MZ971201 | TpiCa1a | Dalu, Meghalaya | Population genetics | | 25.51°N, 90.23° E | India |
| **8** | MZ934367 | R | MZ971202 | TpiCa1a | Darengre, Meghalaya | Population genetics | | 25.20° N, 90.23° E | India |
| **9** | MZ934368 | R | MZ971203 | TpiCa1a | Gambegre, Meghalaya | Population genetics | | 25.43° N, 90.20° E | India |
| **10** | MZ934369 | R | MZ971204 | TpiCa1a | Tikrikilla, Meghalaya | Population genetics | | 25.91° N, 90.15° E | India |
| **11** | MZ956252 | R | MZ971205 | TpiCa1a | Adabari, Assam | Population genetics | | 26.39° N, 90.29° E | India |
| **12** | MZ956253 | R | MZ971182 | TpiCa1a | Anthaibari, Assam | Population genetics | | 26.45° N, 90.02° E | India |
| **13** | MZ956254 | R | MZ971183 | TpiCa1a | Bhutiapara, Assam | Population genetics | | 26.48° N, 90.12° E | India |
| **14** | MZ956255 | R | MZ971184 | TpiCa1a | Jamadarpara, Assam | Population genetics | | 26.35° N, 90.26° E | India |
| **15** | MZ956256 | R | MZ971185 | TpiCa1a | Sijua, Odisha | Population genetics | | 20.22° N, 85.77° E | India |
| **16** | MZ956257 | R | MZ971186 | TpiCa1a | Kesura, Odisha | Population genetics | | 20.27° N, 85.87° E | India |
| **17** | MZ956258 | R | MZ971187 | TpiCa1a | Bankual , Odisha | Population genetics | | 20.25° N, 85.87° E | India |
| **18** | MZ956259 | R | MZ971188 | TpiCa1a | Sarakantara, Odisha | Population genetics | | 20.22° N, 85.79° E | India |
| **19** | MZ956266 | R | MZ971189 | TpiCa2b | Madanpur, West Bengal | Population genetics | | 23.00° N, 88.49° E | India |
| **20** | MZ956267 | R | MZ971190 | TpiCa2b | Chasarhati, West Bengal | Population genetics | | 22.99°N, 88.42° E | India |
| **21** | MZ956268 | R | MZ971191 | TpiCa2b | Simanta, West Bengal | Population genetics | | 22.98° N, 88.42° E | India |
| **22** | MZ956269 | R | MZ971192 | TpiCa2b | Silpanchal, West Bengal | Population genetics | | 22.97° N, 88.45° E | India |
| **23** | MZ956270 | R | MZ971193 | TpiCa2b | Moundouri, West Bengal | Population genetics | | 22.80° N, 88.50° E | India |
| **24** | MZ956271 | R | MZ971194 | TpiCa2b | Jaguli, West Bengal | Population genetics | | 22.92° N, 88.55° E | India |
| **25** | MZ956272 | R | MZ971195 | TpiCa2b | Kanchrapara, West Bengal | Population genetics | | 22.94° N, 88.43° E | India |
| **26** | MZ956273 | R | MZ971196 | TpiCa2b | Haringhata ,West Bengal | Population genetics | | 22.96° N, 88.56° E | India |
| **27** | MZ956260 | R | MZ971197 | TpiCa1a | Kharik, Bihar | Population genetics | | 25.36° N, 87.00° E | India |
| **28** | MZ956261 | R | MZ971198 | TpiCa1a | Narayanpur, Bihar | Population genetics | | 25.40° N, 86.94° E | India |
| **29** | MZ956262 | R | MZ971199 | TpiCa1a | Goradih, Bihar | Population genetics | | 25.14° N, 87.05° E | India |
| **30** | MZ956263 | R | MZ971200 | TpiCa1a | Sabour, Bihar | | Population genetics | 25.24° N, 87.04° E | India |
| **31** | OK178013 | R | OK247557 | TpiCa1a | Kalimpong, West Bengal | | Population genetics | 27.05° N, 88.46° E | India |
| **32** | OK178014 | R | OK247558 | TpiCa1a | Agartala, Tripura | | Population genetics | 23.83° N, 91.28° E | India |
| **33** | OK178015 | R | OK247559 | TpiCa2b | Kishanganj, Bihar | | Population genetics | 26.09° N, 87.94° E | India |
| **34** | OK178016 | R | OK247560 | TpiCa2b | Malda, Bengal | | Population genetics | 25.01° N, 88.14° E | India |
| **35** | OK178017 | R | OK247561 | TpiCa2b | Dhanbad, Jharkhand | | Population genetics | 23.79° N, 86.43° E | India |
| **36** | OK178018 | R | OK247562 | TpiCa2b | Coochbehar, West Bengal | | Population genetics | 26.34° N, 89.44° E | India |
| **37** | OK178019 | R | OK247563 | TpiCa1a | Chandel, Manipur | | Population genetics | 24.32° N, 94.00° E | India |
| **38** | OK178020 | R | OK247564 | TpiCa2b | Mursidabad, West Bengal | | Population genetics | 24.17° N, 88.28° E | India |
| **39** | OK178021 | R | OK247565 | TpiCa2b | Katihar, Bihar | | Population genetics | 25.55° N, 87.55° E | India |
| **40** | OK178261 | C | OK571339 | Tpi-R1a | Alipurduar, West Bengal | | Population genetics | 26.49° N, 89.53° E | India |
| **41** | OK178262 | C | OK571340 | Tpi-R1a | Birpara, West Bengal | | Population genetics | 26.70° N, 89.13° E | India |
| **42** | OK178263 | C | OK571341 | Tpi-R1a | Jalpaiguri, West Bengal | | Population genetics | 26.52° N, 88.71° E | India |

**Table S2:** COIA gene sequences across different geographical region (America, Asia II, Africa and India) used in the present study.

| **CO-I Sequences** | | | |
| --- | --- | --- | --- |
| **America** | **Asia-II** (Excluding India) | **Africa** | **India** (our sequences) |
| 1.KX281221 (North America)  2.U72978 (USA)  3.U72977 (USA)  4.U72976 (USA)  5.U72975 (USA)  6.U72974 (USA)  7.KT809294(Brazil)  8.KT809293(Brazil)  9.KT809292(Brazil)  10.KT809291(Brazil)  11.KT809290(Brazil)  12.KT809289(Brazil)  13.KT809288(Brazil)  14.KT809287(Brazil)  15.KT809286(Brazil)  16.KT809285(Brazil)  17.KT809284(Brazil)  18.KT809283(Brazil)  19.KT809282(Brazil)  20.KT809281(Brazil)  21.KT809280(Brazil)  22.KT809279(Brazil)  23.KT809278(Brazil)  24.KT809277(Brazil)  25.KT809276(Brazil)  26.KT809275(Brazil)  27.KT809274(Brazil)  28.KT809273(Brazil)  29.KT809272(Brazil)  30.KT809271(Brazil)  31.KT809270(Brazil)  32.KT809269(Brazil)  33.KT809268(Brazil)  34.KT809267(Brazil)  35.KT809266(Brazil)  36.KT809265(Brazil)  37.KT809264(Brazil)  38.KT809263(Brazil)  39.KT809262(Brazil)  40.KT809261(Brazil)  41.KT809260(Brazil)  42.KT809259(Brazil)  43.KT809258(Brazil)  44.KT809257(Brazil)  45.KT809256(Brazil)  46.KT809255(Brazil)  47.KT809254(Brazil)  48.KT809253(Brazil)  49.KT809252(Brazil)  50.KT809251(Brazil)  51.KT809250(Brazil)  52.KT809249(Brazil)  53.KT809248(Brazil)  54.KT809247(Brazil)  55.KT809246(Brazil)  56.KT809245(Brazil)  57.KT809244(Brazil)  58.KT809243(Brazil)  59.KT809242(Brazil)  60.KT809241(Brazil)  61.KT809240(Brazil)  62.KT809239(Brazil)  63.KT809238(Brazil)  64.KT809237(Brazil)  65.KT809236(Brazil)  66.KT809235(Brazil)  67.KJ634298(Suriname)  68.KJ634297(Honduras)  69.MK318422(Mexico)  70.MK318420(Mexico)  71.MK318377(Puerto Rico)  72.MK318373(Puerto Rico)  73.MK318372(Mexico)  74.MK318311(Mexico)  75.MK318297 (D.Republic)  76.GU439151(Ontario)  77.GU439150(Puslinch)  78.GU439149(Puslinch)  79.GU439148(Puslinch)  80.GU439147(Puslinch)  81.GU090724(Puslinch)  82.GU090723(Puslinch)  83.GU095403(New Brunswick)  84.GU094756(Puslinch)  85.GU094755(Puslinch)  86.GU094754(Puslinch)  87.KJ388147(Quebec)  88.HM102314(America)  89.KJ641998(Guano)  90.KJ641997(Guano)  91.KF624877(Roraima)  92.KF624876(Roraima)  93.JQ559528(Costa Rica)  94.JQ554012(Costa Rica)  95.JQ572603(Costa Rica)  96.JQ571459(Costa Rica)  97.JQ547900(Costa Rica)  98.JQ577923(Costa Rica)  99.JF854747(Campina Grande)  100.JF854746(Morretes)  101.JF854745(Morretes)  102.JF85474(Campina Grande)  103.JF854743(Morretes)  104.JF854741(Morretes)  105.JF854740(Morretes)  106. HQ964527(Massachusetts)  107. HQ964487(Massachusetts)  108. HQ964486Massachusetts  109.HQ964485(Massachusetts)  110.HQ964443(Massachusetts)  111.HQ964441(Massachusetts)  112.HQ964442(Massachusetts)  113.HQ964440(Massachusetts)  114.HQ964439(Massachusetts)  115.HQ964394(Massachusetts)  116.HQ964393(Massachusetts)  117. HQ964353(Massachusetts)  118. HQ964352(Massachusetts)  119. HQ964351(Massachusetts)  120.GU159435(Costa Rica)  121.GU159434(Costa Rica)  122.GU159433(Costa Rica)  123.GU159432(Costa Rica)  124.GU159431(Costa Rica)  125.GU159430(Costa Rica)  126.GU159429(Costa Rica)  127.GU159428(Costa Rica)  128.GU159427(Costa Rica)  129.GU159426(Costa Rica)  130.GU163698(Costa Rica)  131.HM136602(Florida)  132.HM136601(Florida)  133.HM136600(Florida)  134.HM136599(Florida)  135.HM136598(Florida)  136.HM136597(Florida)  137.HM136596(Florida)  138.HM136595(Florida)  139.HM136594(Florida)  140.HM136593(Florida)  141.HM136592(Florida)  142.HM136591(Florida)  143.HM136590(Florida)  144.HM136589(Florida)  145.HM136588(Florida)  146.HM136587(Florida)  147.HM136586(Florida)  148.HM388081(Bartlesville)  149.GU799699(Bartlesville)  150.MG360803(Ontario)  151.GU658451(Alvaro)  152.JF857952(Yucatan)  153.JF855010(North Carolina)  154.HM406395(Arizona)  155.GU669423(Maryland)  156.HM406394(Arizona)  157.JF855008(Bryson City)  158.HM430360(Arizona)  159.KJ381264(Ontario)  160.KJ393860(Ontario)  161.MG360372(Ontario)  162.KJ389856(Quebec)  163.KF854210 (Florida)  164.KF624877(Roraima) | 1. MT103344(Bangladesh)  2. MT103343(Bangladesh)  3. MT103342(South Korea)  4. MT103341(Viet Nam)  5. MT103340(Viet Nam)  6. MT103339(Viet Nam)  7. MT103338(Viet Nam)  8. MT103336(Viet Nam)  9. MT103335(Viet Nam)  10. MT103334(Viet Nam)  11. MT641270(South Korea)  12. MT641269(South Korea)  13. MT641268(South Korea)  14. LC546868(Japan)  15. LC546867(Japan)  16. LC546866(Japan)  17. LC546865(Japan)  18. LC546864(Japan)  19. LC546863(Japan)  20. LC546862(Japan)  21. LC546861(Japan)  22. LC546860(Japan)  23. LC546859(Japan)  24. LC546858(Japan)  25. LC546857(Japan)  26. LC546856(Japan)  27. LC546855(Japan)  28. LC546854(Japan)  29. LC546853(Japan)  30. LC546852(Japan)  31. LC546851(Japan)  32. LC546850(Japan)  33. LC546849(Japan)  34. LC546848(Japan)  35. LC546847(Japan)  36. LC546846(Japan)  37. MK913648(Vietnam)  38. MK913647(Vietnam)  39. MK913646(Vietnam)  40. MK860942(China)  41. MK860941(China)  42. MK860940(China)  43. MK860939(China)  44. MK860938(China)  45. MK860937(China)  46. MK860936(China)  47. MK860935(China)  48. MK860934(China)  49. MK860933(China)  50.MK860932(China)  51. MK860931(China)  52. MK860930(China)  53. MK860929(China)  54. MK860928(China)  55. MK860927(China)  56. MK860926(China)  57. MK860925(China)  58. MK860924(China)  59. MK860923(China)  60. MK860922(China)  61. MK860921(China)  62. MK860920(China)  63. MK860919(China)  64. MK860918(China)  65. MK713974(Myanmar)  66. MN075831(China)  67. MN075830(China)  68. MK913645(Viet Nam)  69. MT073263(Bangladesh)  70. MT180097(Pakistan)  71. MK790611(China)  72. MT073264(Bangladesh)  73. MT073266(Bangladesh)  74. MT073265(Bangladesh)  75. MN820655(China)  76. MN820654(China) | 1. MF593258(South Africa) 2. MF593257(South Africa) 3. MF593256(South Africa) 4. MF593255(South Africa) 5. MF593254(South Africa) 6. MF593253(South Africa) 7. MF593252(South Africa) 8. MF593251(South Africa) 9. MF593250(South Africa) 10. MF593249(South Africa) 11. MF593248(South Africa) 12. MF593247(South Africa) 13. MF593246(South Africa) 14. MF593245(South Africa) 15. MF593244(South Africa) 16. MF593243(South Africa) 17. MF593242(South Africa) 18. MF593241(South Africa) 19. MK493022(South Africa) 20. MK493021(South Africa) 21. MK493020(South Africa) 22. MK493019(South Africa) 23. MK493018(South Africa) 24. MK493017(South Africa) 25. MK493016(South Africa) 26. MK493015(South Africa) 27. MK493014(South Africa) 28. MK493013(South Africa) 29. MK493012(South Africa) 30. MK493011(South Africa) 31. MT103351(Congo) 32. MT103350(Congo) 33. MT103349(Congo) 34. MT103348(Tanzania) 35. MT103347(Mazowe) 36. MT103346(Harare) 37. KX580619(Nigeria) 38. KX580618(Nigeria) 39. KX580617(Nigeria) 40. KX580616(Nigeria) 41. KX580615(Sao-Tome) 42. KX580614(Sao-Tome) 43. MT641267(Uganda) 44. MF278659(Tanzania) 45. MF278658(Tanzania) 46. MF278657(Tanzania) 47. MH190448(Kenya) 48. MH190447(Kenya) 49. MH190446(Kenya) 50. MH190445(Kenya) 51. MH190444(Kenya) 52. KY472255(Ghana) 53. KY472254(Ghana) 54. KY472253(Ghana) 55. KY472252(Ghana) 56. KY472251(Ghana) 57. KY472250(Ghana) 58. KY472249(Ghana) 59. KY472248(Ghana) 60. KY472245(Ghana) 61. KY472244(Ghana) 62. KY472242(Ghana) 63. KY472241(Ghana) 64. KY472240(Ghana) 65. MG993205(Malawi) 66. MF197867(Uganda) 67. MK493006(Kenya) 68. MK493000(Kenya) 69. MK492996(Kenya) 70. MK493010(Kenya) 71. MK493009(Kenya) 72. MK493008(Kenya) 73. MK493007(Kenya) 74. MK493004(Kenya) 75. MK493003(Kenya) 76. MK493002(Kenya) 77. MK493001(Kenya) 78. MK492999(Kenya) 79. MK492998(Kenya) 80. MK492997(Kenya) 81. MK492995(Kenya) 82. MK492994(Kenya) 83. MK492993(Kenya) 84. MK492992(Kenya) 85. MK492991(Kenya) 86. MK492990(Kenya) 87. MK492989(Kenya) 88. MK492988(Kenya) 89. MK492987(Kenya) 90. MK492986(Kenya) 91. MK492985(Kenya) 92. MK492984(Kenya) 93. MK492983(Kenya) 94. MK492982(Kenya) 95. MK492981(Kenya) 96. MK492979(Kenya) 97. MK492978(Kenya) 98. MK492977(Kenya) 99. MK492976(Kenya) 100. MK492975(Kenya) 101. MK492973(Kenya) 102. MK492972(Kenya) 103. MK492971(Kenya) 104. MK492970(Kenya) 105. MK492969(Kenya) 106. MK492968(Kenya) 107. MK492967(Kenya) 108. MK492966(Kenya) 109. MK492965(Kenya) 110. MK492964(Kenya) 111. MK492963(Kenya) 112. MK492962(Kenya) 113. MK492961(Kenya) 114. MK492960(Kenya) 115. MK492959(Kenya) 116. MK492958(Kenya) 117. MK492957(Kenya) 118. MK492956(Kenya) 119. MK492955(Kenya) 120. MK492954(Kenya) 121. MK492953(Kenya) 122. MK492952(Kenya) 123. MK492951(Kenya) 124. MK492950(Kenya) 125. MK492949(Kenya) 126. MK492948(Kenya) 127. MK492947(Kenya) 128. MK492946(Kenya) 129. MK492945(Kenya) 130. MK492944(Kenya) 131. MK492943(Kenya) 132. MK492942(Kenya) 133. MK492939(Kenya) 134. MK492938(Kenya) 135. MK492936(Kenya) 136. MK492933(Kenya) 137. MK492930(Kenya) 138. MK492929(Kenya) 139. MF197868(Uganda) 140. MK492980(Kenya) 141. MK492941(Kenya) 142. MK492940(Kenya) 143. MK492937(Kenya) 144. MK492935(Kenya) 145. MK492934(Kenya) 146. MK492932(Kenya) 147. MK492931(Kenya) 148. MK493005(Kenya) 149. MK492974(Kenya) | 1. MZ427465 (India)  2. MZ462994 (India)  3. MZ488270 (India)  4. MZ462066 (India)  5. MZ429433 (India)  6. MZ577176 (India)  7. MZ934366 (India)  8. MZ934367 (India)  9. MZ934368 (India)  10. MZ934369 (India)  11. MZ956252 (India)  12. MZ956253 (India)  13. MZ956254 (India)  14. MZ956255 (India)  15. MZ956256 (India)  16. MZ956257 (India)  17. MZ956258 (India)  18. MZ956259 (India)  19. MZ956266 (India)  20. MZ956267 (India)  21. MZ956268 (India)  22. MZ956269 (India)  23. MZ956270 (India)  24. MZ956271 (India)  25. MZ956272 (India)  26. MZ956273 (India)  27. MZ956260 (India)  28. MZ956261 (India)  29. MZ956262 (India)  30. MZ956263 (India)  31. OK178013 (India)  32. OK178014 (India)  33. OK178015 (India)  34. OK178016 (India)  35. OK178017 (India)  36. OK178018 (India)  37. OK178019 (India)  38. OK178020 (India)  39. OK178021 (India)  40. OK178261 (India)  41. OK178262 (India)  42. OK178263 (India) |

**Table S3**: Summary of genetic diversity of FAW populations analysed on the basis of partial mt-COIA gene from four different geographical location i.e., India, America, Africa and Asia-II. (^**^P<0.02) (^*^P<0.05).

|  | **India** | **America** | **Africa** | **Asia II** | **Total** |
| --- | --- | --- | --- | --- | --- |
| **No of Sequences** | 42 | 164 | 149 | 76 | 431 |
| **No of sites** | 459 | 459 | 452 | 460 | 450 |
| **No of Polymorphic sites(S)** | 14 | 46 | 8 | 12 | 53 |
| **No of Mutation** | 15 | 50 | 8 | 12 | 57 |
| **No of haplotypes (h)** | 5 | 35 | 4 | 5 | 41 |
| **Haplotype diversity(Hd)** | 0.444 | 0.746 | 0.285 | 0.383 | 0.541 |
| **Nucleotide diversity(π)** | 0.00753 | 0.00061 | 0.00180 | 0.000348 | 0.00055 |
| **Fu’s Fs statistic** | -3.054 | -14.059 | 4.758 | 5.224 | -18.266 |
| **Fu and Li’s D* test statistic** | -1.567*(P<0.05) | -5.454**(P<0.02) | 0.378(P>0.10) | -0.285(P>0.10) | -6.272**(P<0.02) |
| **Fu and Li’s F* test statistic** | -0.937(P>0.10) | -4.5429**(P<0.02) | 0.608(P>0.10) | 0.541(P>0.10) | -4.972**(P<0.02) |
| **Tajima’s D** | -0.743 | -1.565 | 0.757 | 0.776 | -1.711 |

**Table S4**: Comparison between genetic diversity of FAW sister strains in India. (^*^P<0.05).

|  | **COI‘R’** | **COI‘C’** |
| --- | --- | --- |
| **No of Sequences** | 39 | 3 |
| **No of sites** | 537 | 655 |
| **No of Polymorphic sites(S)** | 5 | 1 |
| **No of Mutation** | 5 | 1 |
| **No of haplotypes (h)** | 3 | 2 |
| **Haplotype diversity (Hd)** | 0.235 | 0.667 |
| **Nucleotide diversity** | 0.00712 | 0.04167 |
| **Fu’s Fs statistic** | -0.242 | 0.540 |
| **Fu and Li’s D* test statistic** | -2.56927*(P<0.05) | 1.63299 |
| **Fu and Li’s F* test statistic** | -2.69186*(P<0.05) | 1.27657 |
| **Tajima’s D** | -1.70661* | 1.63299 |

**Table S5:** Result of AMOVA analysis among the different FAW geographical groups

| **Groups** | **Source of Variation** | **Df** | **SS** | **Variance Component** | **Total Variance (%)** | **Fixation Index (F_ST_)** | **P value** |
| --- | --- | --- | --- | --- | --- | --- | --- |
| **All** | Between groups | 3 | 652.271 | 1.445 | 15.444 | 0.154 | 0.001 |
|  | Within groups | 431 | 3409.474 | 7.911 | 84.555 |  |  |
|  | Total | 434 | 4061.745 | 9.356 |  |  |  |
| **America Vs India** | Between groups | 1 | 715.243 | 3.030 | 68.924 | 0.689 | 0.001 |
|  | Within groups | 206 | 3687.036 | 1.360 | 31.075 |  |  |
|  | Total | 207 | 4402.280 | 4.396 |  |  |  |
| **Africa and India** | Between groups | 1 | 2783.276 | 2.151 | 9.044 | 0.090 | 0.005 |
|  | Within groups | 191 | 78237.489 | 0.216 | 90.955 |  |  |
|  | Total | 192 | 81020.766 | 2.378 |  |  |  |
| **India and Asia-II** | Between groups | 1 | 18.023 | 0.108 | 29.870 | 0.298 | 0.001 |
|  | Within groups | 118 | 393.898 | 0.253 | 70.129 |  |  |
|  | Total | 119 | 411.921 | 0.361 |  |  |  |

**Table S6**: Susceptibility of *Spodoptera frugiperda* field populations to three insecticides.

| **Insecticide** | **Location** | **LC_50_ (mg/L)** | **95% C.L.** | **RR at LC_50_** | **Slope±SE** | **χ^2^(*p*)** |
| --- | --- | --- | --- | --- | --- | --- |
| **Chlorantraniliprole** | **Laboratory** | 17.60 | 4.61-43.16 | 1.00 | 0.56±0.12 | 5.11  (0.16) |
|  | **Assam** | 74.09 | 29.16-213.31 | 4.21 | 0.54±0.13 | 5.34  (0.15) |
|  | **Bihar** | 46.15 | 16.52-119.72 | 2.62 | 0.54±0.12 | 4.97  (0.17) |
|  | **Meghalaya** | 34.70 | 12.52-81.23 | 1.97 | 0.59±0.12 | 5.72  (0.13) |
|  | **Odisha** | 58.55 | 20.30-176.24 | 3.33 | 0.5±0.12 | 5.31  (0.15) |
|  | **West Bengal** | 89.05 | 36.96-218.16 | 5.06 | 0.61±0.13 | 5.54  (0.14) |
| **Spinetoram** | **Laboratory** | 23.87 | 7.31-53.38 | 1.00 | 0.61±0.13 | 2.44  (0.49) |
|  | **Assam** | 131.28 | 30.87-202.21 | 5.5 | 0.58±0.14 | 1.07  (0.78) |
|  | **Bihar** | 46.59 | 13.80-131.03 | 1.95 | 0.49±0.13 | 3.04  (0.39) |
|  | **Meghalaya** | 49.18 | 19.93-110.33 | 2.06 | 0.63±0.14 | 5.97  (0.11) |
|  | **Odisha** | 65.75 | 30.17-142.22 | 2.75 | 0.69±0.14 | 3.18  (0.36) |
|  | **West Bengal** | 53.45 | 22.47-118.27 | 2.24 | 0.65±0.14 | 2.91  (0.41) |
| **Cypermethrin** | **Laboratory** | 39.98 | 16.93-78.88 | 1.00 | 0.73±0.15 | 3.09  (0.38) |
|  | **Assam** | 142.87 | 67.66-436.09 | 3.57 | 0.64±0.16 | 2.18  (0.54) |
|  | **Bihar** | 109.49 | 51.42-292.54 | 2.74 | 0.65±0.16 | 1.54  (0.67) |
|  | **Meghalaya** | 77.16 | 38.59-161.09 | 1.93 | 0.75±0.16 | 4.87  (0.18) |
|  | **Odisha** | 149.41 | 72.17-446.69 | 3.74 | 0.67±0.16 | 4.54  (0.21) |
|  | **West Bengal** | 105.75 | 52.91-248.93 | 2.65 | 0.72±0.16 | 3.22  (0.36) |

**Table S7:** Primers used in the current study. The primer name, PCR type, primer sequences and the annealing temperature are listed in the table below (*EF: Elongation factor).

| **Primer name** | **PCR type** | **Primer sequences (5′→3′)** | | **Annealing temperature (°C)** |
| --- | --- | --- | --- | --- |
|  |  | **Primer forward** | **Primer reverse** |  |
| **1. CO-1** | **Conventional PCR** | **GGTCAACAAATCATAAAGATATTGG** | **TAAACTTCAGGGTGACCAAAAAATCA** | 54 |
| **2. Tpi** | **Conventional PCR** | **CCGGACTGAAGGTTATCGCTTG** | **GCGGAAGCATTCGCTGACAACC** | 56 |
| **3. 1950 (GST)** | **qRT- PCR** | **CGATTTGGGCACCTTGTATC** | **TGTGGCCCTCTAGGAAAGTG** | 60 |
| **4. 3423 (GST)** | **qRT- PCR** | **GGTTTGGTAGAGGCAAGCAA** | **GCCGCAACATACTCCTTGAC** | 60 |
| **5. 801 (GST)** | **qRT- PCR** | **AGCCGGTGAACATCTCACTC** | **TCCTTTCCGTAGGTGTCAGC** | 60 |
| **6. 968 (GST)** | **qRT- PCR** | **CGTCCTATGCGGTTTAGTGG** | **ACTCCTTGACCCCGGTAGAC** | 60 |
| **7. 9131 (CYP)** | **qRT- PCR** | **TACGGACACAGTGGCAAGAC** | **CTGGGTTCATCGCTAGTTGG** | 60 |
| **8. 9360 (CYP)** | **qRT- PCR** | **GCCAGATTAGGACAGATGCAG** | **CCAACAAAACCTTCGGAGAC** | 60 |
| **9. EF** | **qRT- PCR** | **TCGCTGTGGGTGTAATCAAG** | **GCTACTTCTTGCCCTTGGTG** | 60 |

1. # Snigdha Samanta and Mritunjoy Barman have equally contributed to this work. [↑](#footnote-ref-1)
